# Supplementary material for: A new family of structurally conserved fungal effectors displays epistatic interactions with plant resistance proteins
Source: PLoS Pathog. 2022 Jul 6;18(7):e1010664. doi: 10.1371/journal.ppat.1010664 (PMC9292093; doi:10.1371/journal.ppat.1010664)
Supplement: S1 Fig — (A) Size-exclusion chromatogram (Superdex 75 16/60 column; GE Healthcare); elution with 20 mM Tris pH 8.0, 300 mM NaCl, 5% glycerol. (B) SDS-PAGE analysis of the peak fractions of the gel-filtration step. Protein sizes are shown in kDa. (PDF) [file ppat.1010664.s001.pdf]

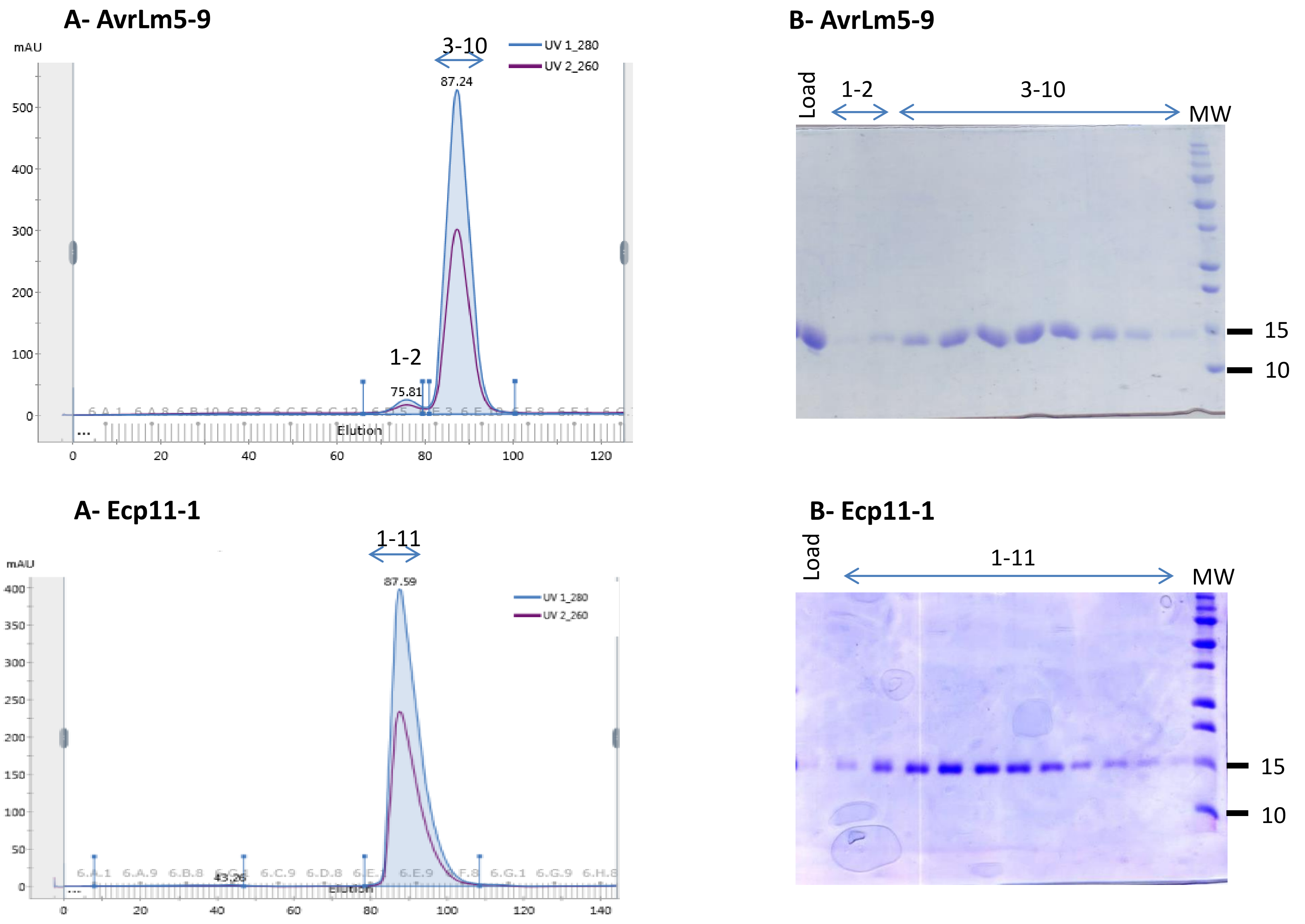

Fig S1. Purification of the recombinant AvrLm5-9 and Ecp11-1 proteins.
(A) Size-exclusion chromatogram (Superdex 75 16/60 column; GE Healthcare); elution with 20 mM Tris pH 8.0, 300 mM NaCl, 5 % glycerol. (B) SDS-PAGE gel analysis of the peak fractions of the gel-filtration step. Protein sizes are shown in kDa.
