# Supplementary material for: A new family of structurally conserved fungal effectors displays epistatic interactions with plant resistance proteins
Source: PLoS Pathog. 2022 Jul 6;18(7):e1010664. doi: 10.1371/journal.ppat.1010664 (PMC9292093; doi:10.1371/journal.ppat.1010664)
Supplement: S2 Fig — The multiple sequence alignment generated in Fig 5 was used to generate a diversity tree using the Neighbor-joining method. Branch supports are based on 1000 bootstraps and horizontal branch length reflects sequence divergence. Cg, Colletotrichum gloeosporioides; Ch, Colletotrichum higginsianum; Co, Colletotrichum orbiculare; Cc, Corynespora cassiicola; Ff, Fulvia fulva; Lbb, Leptosphaeria biglobosa ‘brassicae’; Lbt, Leptosphaeria biglobosa ‘thlaspii’; Lmb, Leptosphaeria maculans ‘brassicae’; Lml, Leptosphaeria maculans ‘lepidii’; Mp, Macrophomina phaseolina; Pt, Pyrenophora teres; Ptr, Pyrenophora tritici-repentis; Sl, Stemphylium lycopersici. (PDF) [file ppat.1010664.s002.pdf]

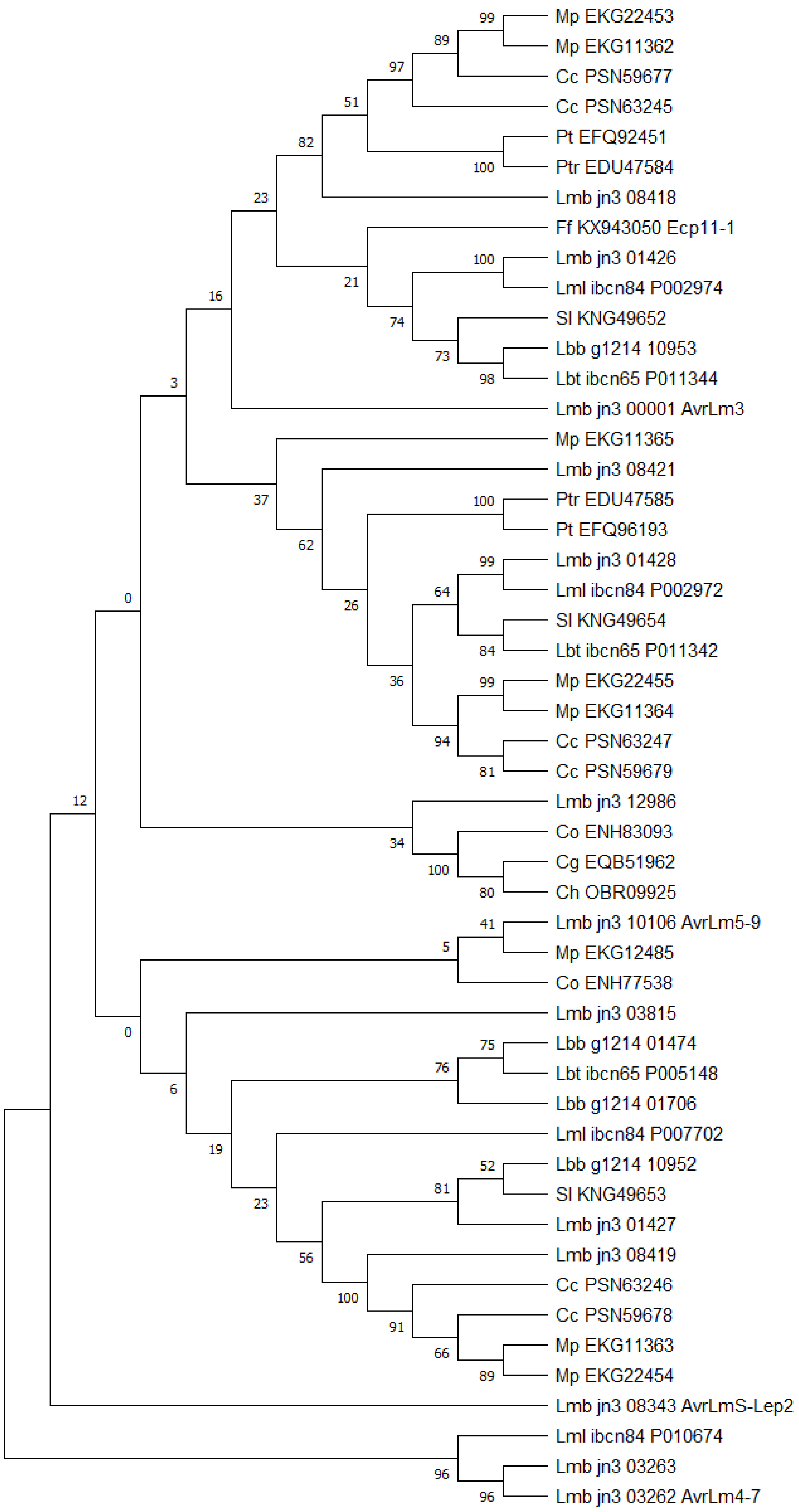

Fig S2. Diversity of LARS effector structural analogues identified by HMM analyses in *L. maculans* 'brassicae' and other phytopathogenic fungi.
The multiple sequence alignment generated in Figure 5 was used to generate a diversity tree using the Neighbor-joining method. Branch supports are based on 1000 bootstraps and horizontal branch length reflects sequence divergence.
Cg, *Colletotrichum gloeosporioides*; Ch, *Colletotrichum higginsianum*; Co, *Colletotrichum orbiculare;* Cc, *Corynespora cassiicola*; Ff, *Fulvia fulva*; Lbb, *Leptosphaeria biglobosa* 'brassicae'; Lbt, *Leptosphaeria biglobosa* 'thlaspii'; Lmb, *Leptosphaeria maculans* 'brassicae'; Lml, *Leptosphaeria maculans* 'lepidii'; Mp, *Macrophomina phaseolina*; Pt, *Pyrenophora teres;* Ptr, *Pyrenophora tritici-repentis*; Sl, *Stemphylium lycopersici*
