# Supplementary material for: A new family of structurally conserved fungal effectors displays epistatic interactions with plant resistance proteins
Source: PLoS Pathog. 2022 Jul 6;18(7):e1010664. doi: 10.1371/journal.ppat.1010664 (PMC9292093; doi:10.1371/journal.ppat.1010664)
Supplement: S3 Fig — The 3D structures of the different LARS effectors found by the HMM search were modeled using AlphaFold2 [27]. Cysteines are presented as sticks and colored in red. No reliable models could be obtained for Lmb_jn3_08343 or Lmb_jn3_12986. (PPTX) [file ppat.1010664.s003.pptx]

## Slide 1
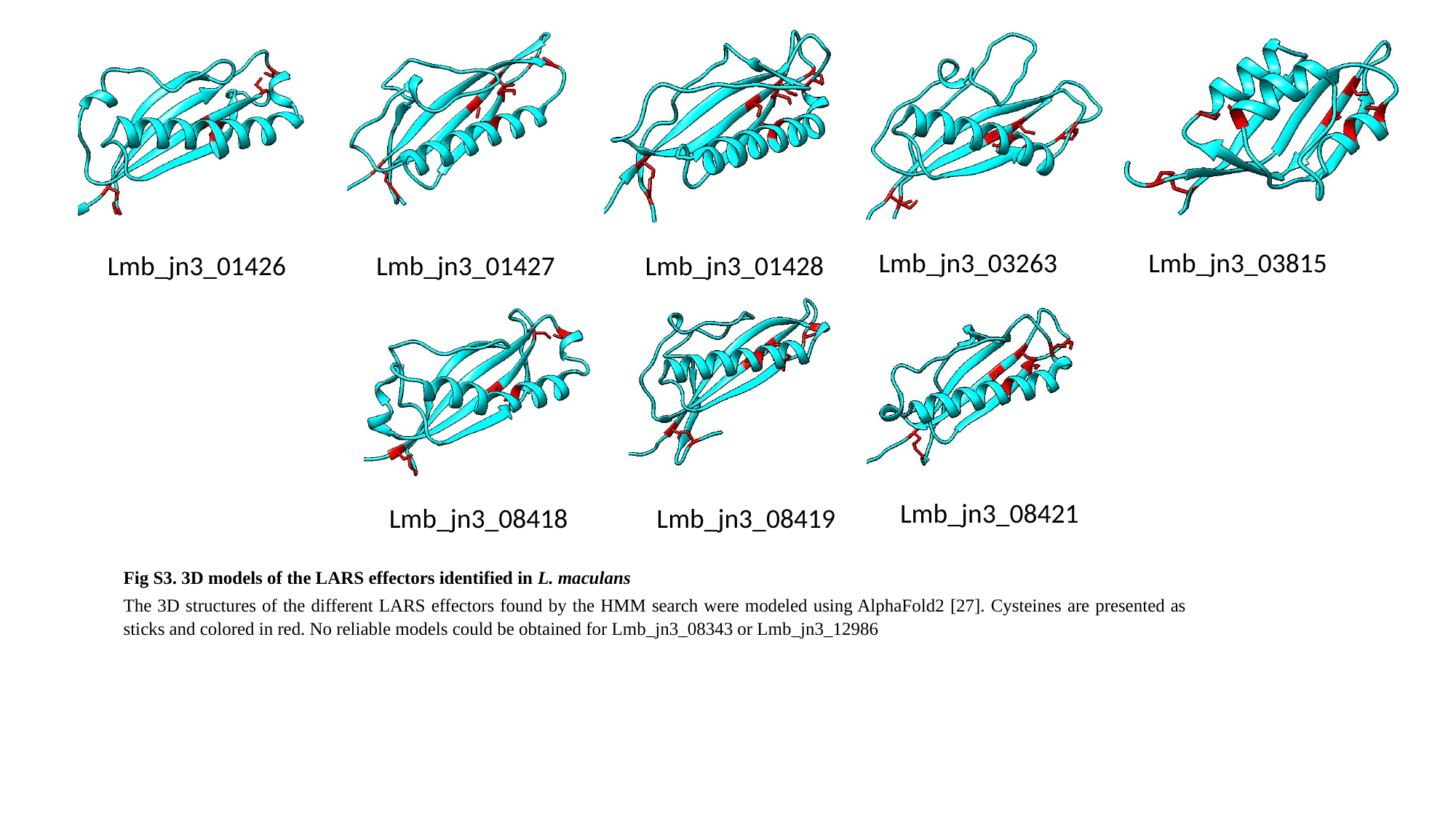

Lmb_jn3_03263
Lmb_jn3_03815
Lmb_jn3_01426
Lmb_jn3_01427
Lmb_jn3_01428
Lmb_jn3_08421
Lmb_jn3_08418
Lmb_jn3_08419
Fig S3. 3D models of the LARS effectors identified in L. maculans
The 3D structures of the different LARS effectors found by the HMM search were modeled using AlphaFold2 [27]. Cysteines are presented as sticks and colored in red. No reliable models could be obtained for Lmb_jn3_08343 or Lmb_jn3_12986
