# Supplementary material for: A new family of structurally conserved fungal effectors displays epistatic interactions with plant resistance proteins
Source: PLoS Pathog. 2022 Jul 6;18(7):e1010664. doi: 10.1371/journal.ppat.1010664 (PMC9292093; doi:10.1371/journal.ppat.1010664)
Supplement: S4 Fig — Wild type isolates G06-E107 (A3a4a7), JN3 (a3A4A7) and JN2 (a3a4A7), as well as G06-E107 transformants carrying AvrLm4-7 alleles with mutations at amino acids R100, F102, S112 and / or G120 were inoculated onto cotyledons of cultivars carrying Rlm3 (15.22.4.1), Rlm7 (15.23.4.1) or Rlm4 (Pixel). Pathogenicity was measured 10 and 14 days post-inoculation (DPI). Results are expressed as a mean scoring using the IMASCORE rating comprising six infection classes (IC), where IC1 to IC3 correspond to resistance, and IC4 to IC6 to susceptibility [50]. Error bars indicate the standard deviation of technical replicates. (PPTX) [file ppat.1010664.s004.pptx]

## Slide 1
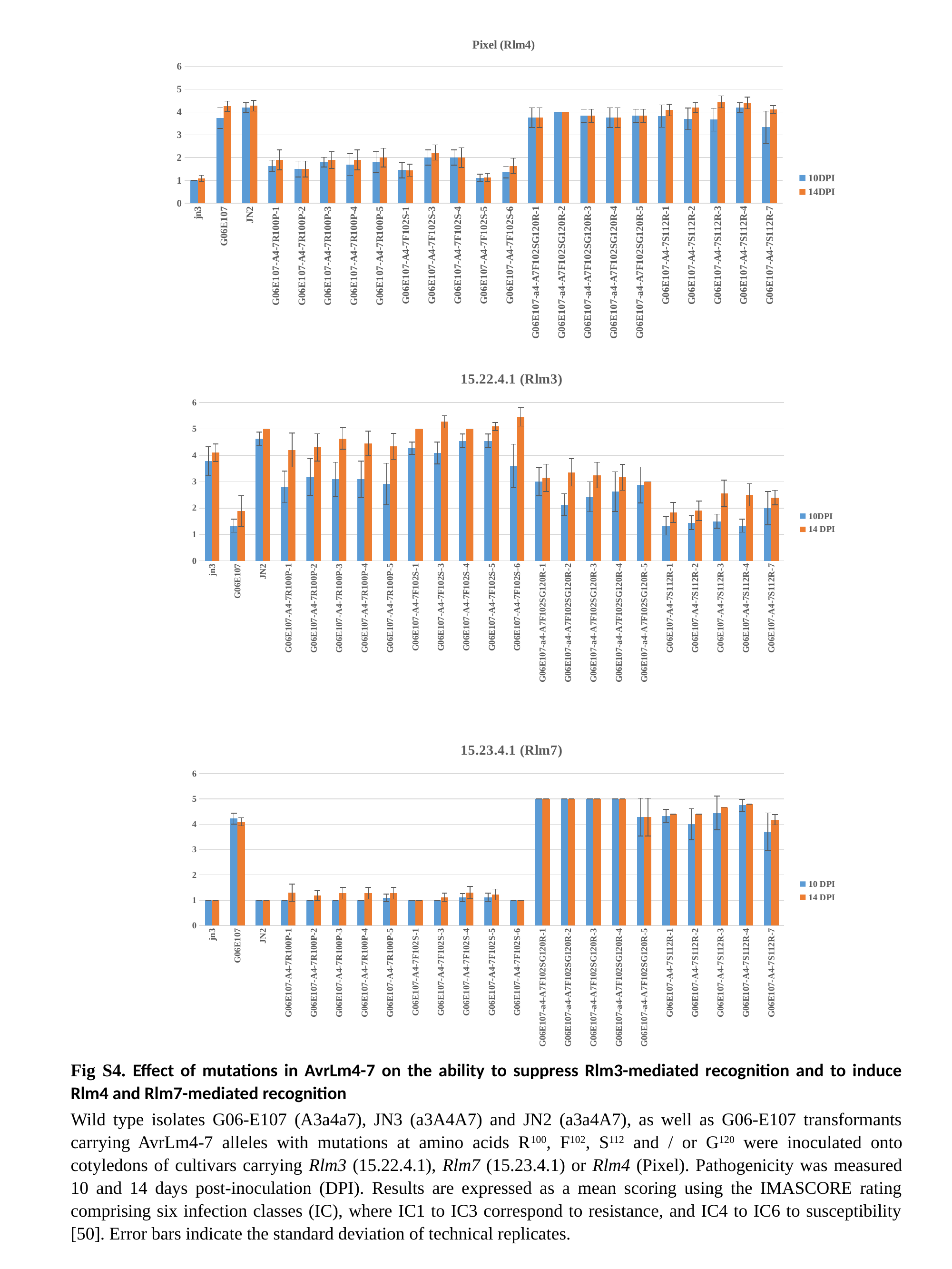

### Chart: Pixel (Rlm4)
| Category | | |
|---|---|---|
| jn3 | 1.0 | 1.0833333333333333 |
| G06E107 | 3.727272727272727 | 4.25 |
| JN2 | 4.2 | 4.2727272727272725 |
| G06E107-A4-7R100P-1 | 1.6363636363636365 | 1.9 |
| G06E107-A4-7R100P-2 | 1.5 | 1.5 |
| G06E107-A4-7R100P-3 | 1.8 | 1.9 |
| G06E107-A4-7R100P-4 | 1.7 | 1.9 |
| G06E107-A4-7R100P-5 | 1.8 | 2.0 |
| G06E107-A4-7F102S-1 | 1.4545454545454546 | 1.4444444444444444 |
| G06E107-A4-7F102S-3 | 2.0 | 2.2222222222222223 |
| G06E107-A4-7F102S-4 | 2.0 | 2.0 |
| G06E107-A4-7F102S-5 | 1.1111111111111112 | 1.125 |
| G06E107-A4-7F102S-6 | 1.3636363636363635 | 1.6363636363636365 |
| G06E107-a4-A7F102SG120R-1 | 3.75 | 3.75 |
| G06E107-a4-A7F102SG120R-2 | 4.0 | 4.0 |
| G06E107-a4-A7F102SG120R-3 | 3.8333333333333335 | 3.8333333333333335 |
| G06E107-a4-A7F102SG120R-4 | 3.75 | 3.75 |
| G06E107-a4-A7F102SG120R-5 | 3.8333333333333335 | 3.8333333333333335 |
| G06E107-A4-7S112R-1 | 3.8181818181818183 | 4.083333333333333 |
| G06E107-A4-7S112R-2 | 3.7 | 4.2 |
| G06E107-A4-7S112R-3 | 3.6666666666666665 | 4.444444444444445 |
| G06E107-A4-7S112R-4 | 4.2 | 4.4 |
| G06E107-A4-7S112R-7 | 3.3333333333333335 | 4.111111111111111 |
### Chart: 15.22.4.1 (Rlm3)
| Category | | |
|---|---|---|
| jn3 | 3.7777777777777777 | 4.1 |
| G06E107 | 1.3333333333333333 | 1.8888888888888888 |
| JN2 | 4.625 | 5.0 |
| G06E107-A4-7R100P-1 | 2.8 | 4.2 |
| G06E107-A4-7R100P-2 | 3.1818181818181817 | 4.3 |
| G06E107-A4-7R100P-3 | 3.090909090909091 | 4.636363636363637 |
| G06E107-A4-7R100P-4 | 3.090909090909091 | 4.454545454545454 |
| G06E107-A4-7R100P-5 | 2.9166666666666665 | 4.333333333333333 |
| G06E107-A4-7F102S-1 | 4.2727272727272725 | 5.0 |
| G06E107-A4-7F102S-3 | 4.090909090909091 | 5.2727272727272725 |
| G06E107-A4-7F102S-4 | 4.545454545454546 | 5.0 |
| G06E107-A4-7F102S-5 | 4.545454545454546 | 5.090909090909091 |
| G06E107-A4-7F102S-6 | 3.6 | 5.454545454545454 |
| G06E107-a4-A7F102SG120R-1 | 3.0 | 3.15 |
| G06E107-a4-A7F102SG120R-2 | 2.125 | 3.35 |
| G06E107-a4-A7F102SG120R-3 | 2.4285714285714284 | 3.25 |
| G06E107-a4-A7F102SG120R-4 | 2.625 | 3.1666666666666665 |
| G06E107-a4-A7F102SG120R-5 | 2.875 | 3.0 |
| G06E107-A4-7S112R-1 | 1.3333333333333333 | 1.8333333333333333 |
| G06E107-A4-7S112R-2 | 1.4444444444444444 | 1.9 |
| G06E107-A4-7S112R-3 | 1.5 | 2.5555555555555554 |
| G06E107-A4-7S112R-4 | 1.3333333333333333 | 2.5 |
| G06E107-A4-7S112R-7 | 2.0 | 2.4 |
### Chart: 15.23.4.1 (Rlm7)
| Category | | |
|---|---|---|
| jn3 | 1.0 | 1.0 |
| G06E107 | 4.222222222222222 | 4.1 |
| JN2 | 1.0 | 1.0 |
| G06E107-A4-7R100P-1 | 1.0 | 1.3 |
| G06E107-A4-7R100P-2 | 1.0 | 1.1818181818181819 |
| G06E107-A4-7R100P-3 | 1.0 | 1.2727272727272727 |
| G06E107-A4-7R100P-4 | 1.0 | 1.2727272727272727 |
| G06E107-A4-7R100P-5 | 1.0909090909090908 | 1.2727272727272727 |
| G06E107-A4-7F102S-1 | 1.0 | 1.0 |
| G06E107-A4-7F102S-3 | 1.0 | 1.1111111111111112 |
| G06E107-A4-7F102S-4 | 1.1 | 1.3 |
| G06E107-A4-7F102S-5 | 1.1111111111111112 | 1.2222222222222223 |
| G06E107-A4-7F102S-6 | 1.0 | 1.0 |
| G06E107-a4-A7F102SG120R-1 | 5.0 | 5.0 |
| G06E107-a4-A7F102SG120R-2 | 5.0 | 5.0 |
| G06E107-a4-A7F102SG120R-3 | 5.0 | 5.0 |
| G06E107-a4-A7F102SG120R-4 | 5.0 | 5.0 |
| G06E107-a4-A7F102SG120R-5 | 4.285714285714286 | 4.285714285714286 |
| G06E107-A4-7S112R-1 | 4.333333333333333 | 4.4 |
| G06E107-A4-7S112R-2 | 4.0 | 4.4 |
| G06E107-A4-7S112R-3 | 4.444444444444445 | 4.67 |
| G06E107-A4-7S112R-4 | 4.75 | 4.8 |
| G06E107-A4-7S112R-7 | 3.7 | 4.181818181818182 |Fig S4. Effect of mutations in AvrLm4-7 on the ability to suppress Rlm3-mediated recognition and to induce Rlm4 and Rlm7-mediated recognition
Wild type isolates G06-E107 (A3a4a7), JN3 (a3A4A7) and JN2 (a3a4A7), as well as G06-E107 transformants carrying AvrLm4-7 alleles with mutations at amino acids R100, F102, S112 and / or G120 were inoculated onto cotyledons of cultivars carrying Rlm3 (15.22.4.1), Rlm7 (15.23.4.1) or Rlm4 (Pixel). Pathogenicity was measured 10 and 14 days post-inoculation (DPI). Results are expressed as a mean scoring using the IMASCORE rating comprising six infection classes (IC), where IC1 to IC3 correspond to resistance, and IC4 to IC6 to susceptibility [50]. Error bars indicate the standard deviation of technical replicates.
