# Supplementary material for: A new family of structurally conserved fungal effectors displays epistatic interactions with plant resistance proteins
Source: PLoS Pathog. 2022 Jul 6;18(7):e1010664. doi: 10.1371/journal.ppat.1010664 (PMC9292093; doi:10.1371/journal.ppat.1010664)
Supplement: S5 Fig — Wild type isolates Nz-T4 (a1a3a4a7), JN3 (A1a3A4A7) and 19.4.24 (A1A3a4a7), as well as Nz-T4 transformants carrying ECP11-1 or AvrLm3 were inoculated onto cotyledons of three cultivars carrying Rlm3 (15.22.4.1, Grizzly and Columbus), Rlm7 (15.23.4.1) or Rlm4 (Pixel). 15.23.4.1 is a sister line of 15.22.4.1 issued from individual plants from cv. Rangi, carrying Rlm7 instead of Rlm3. Pathogenicity was measured 15 days post-inoculation. Results are expressed as a mean scoring using the IMASCORE rating comprising six infection classes (IC), where IC1 to IC3 correspond to resistance, and IC4 to IC6 to susceptibility [50]. Error bars indicate the standard deviation of technical replicates. (PPTX) [file ppat.1010664.s005.pptx]

## Slide 1
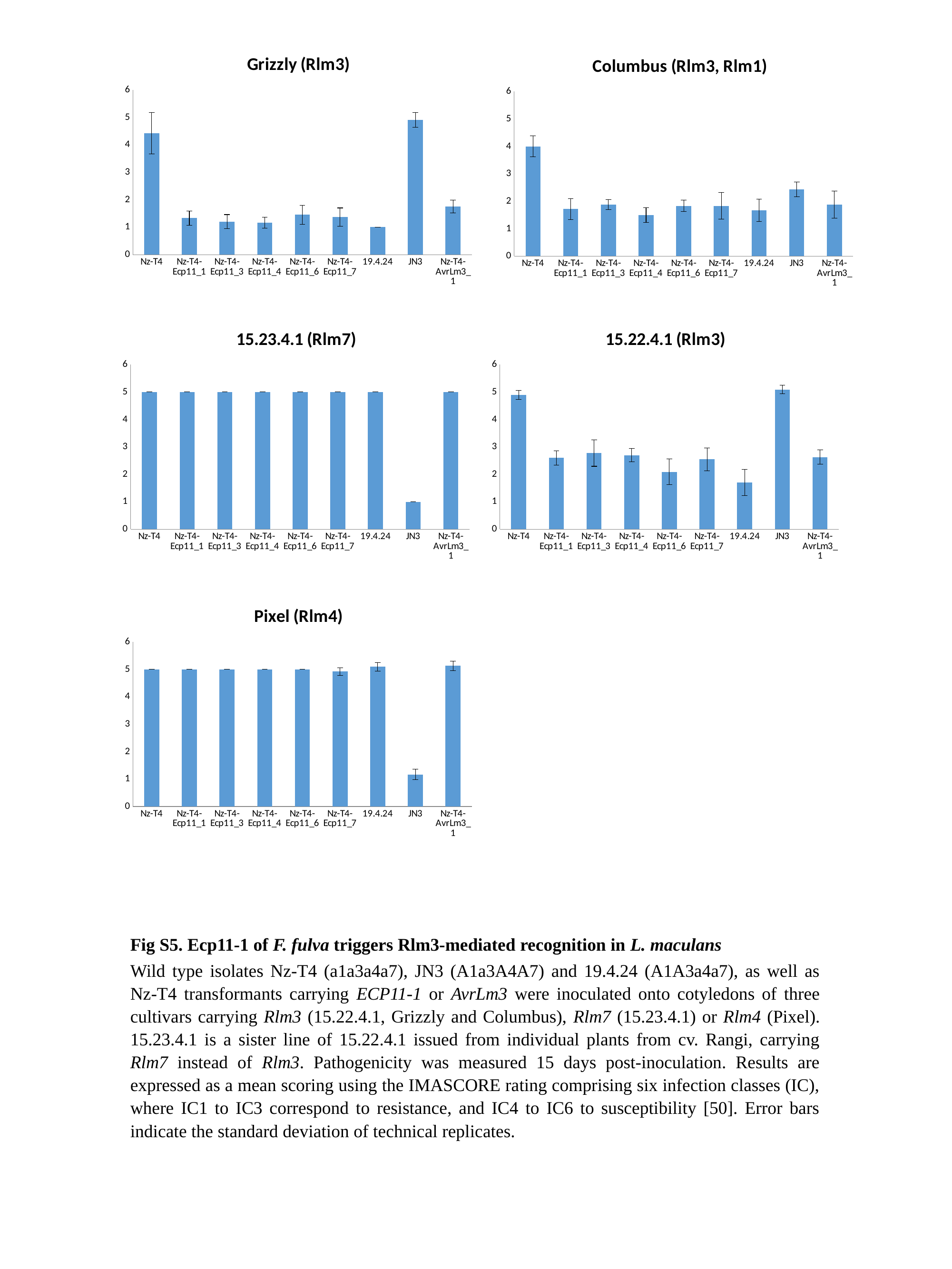

### Chart: Grizzly (Rlm3)
| Category | Nz-T4 |
|---|---|
| Nz-T4 | 4.428571428571429 |
| Nz-T4-Ecp11_1 | 1.3333333333333333 |
| Nz-T4-Ecp11_3 | 1.2 |
| Nz-T4-Ecp11_4 | 1.1666666666666667 |
| Nz-T4-Ecp11_6 | 1.4545454545454546 |
| Nz-T4-Ecp11_7 | 1.3636363636363635 |
| 19.4.24 | 1.0 |
| JN3 | 4.909090909090909 |
| Nz-T4-AvrLm3_1 | 1.75 |
### Chart: Columbus (Rlm3, Rlm1)
| Category | Nz-T4 |
|---|---|
| Nz-T4 | 4.0 |
| Nz-T4-Ecp11_1 | 1.7142857142857142 |
| Nz-T4-Ecp11_3 | 1.875 |
| Nz-T4-Ecp11_4 | 1.5 |
| Nz-T4-Ecp11_6 | 1.8333333333333333 |
| Nz-T4-Ecp11_7 | 1.8333333333333333 |
| 19.4.24 | 1.6666666666666667 |
| JN3 | 2.4285714285714284 |
| Nz-T4-AvrLm3_1 | 1.875 |
### Chart: 15.23.4.1 (Rlm7)
| Category | Nz-T4 |
|---|---|
| Nz-T4 | 5.0 |
| Nz-T4-Ecp11_1 | 5.0 |
| Nz-T4-Ecp11_3 | 5.0 |
| Nz-T4-Ecp11_4 | 5.0 |
| Nz-T4-Ecp11_6 | 5.0 |
| Nz-T4-Ecp11_7 | 5.0 |
| 19.4.24 | 5.0 |
| JN3 | 1.0 |
| Nz-T4-AvrLm3_1 | 5.0 |
### Chart: 15.22.4.1 (Rlm3)
| Category | Nz-T4 |
|---|---|
| Nz-T4 | 4.888888888888889 |
| Nz-T4-Ecp11_1 | 2.6 |
| Nz-T4-Ecp11_3 | 2.7777777777777777 |
| Nz-T4-Ecp11_4 | 2.7 |
| Nz-T4-Ecp11_6 | 2.090909090909091 |
| Nz-T4-Ecp11_7 | 2.5454545454545454 |
| 19.4.24 | 1.7 |
| JN3 | 5.090909090909091 |
| Nz-T4-AvrLm3_1 | 2.625 |
### Chart: Pixel (Rlm4)
| Category | Nz-T4 |
|---|---|
| Nz-T4 | 5.0 |
| Nz-T4-Ecp11_1 | 5.0 |
| Nz-T4-Ecp11_3 | 5.0 |
| Nz-T4-Ecp11_4 | 5.0 |
| Nz-T4-Ecp11_6 | 5.0 |
| Nz-T4-Ecp11_7 | 4.916666666666667 |
| 19.4.24 | 5.090909090909091 |
| JN3 | 1.1666666666666667 |
| Nz-T4-AvrLm3_1 | 5.125 |Fig S5. Ecp11-1 of F. fulva triggers Rlm3-mediated recognition in L. maculans
Wild type isolates Nz-T4 (a1a3a4a7), JN3 (A1a3A4A7) and 19.4.24 (A1A3a4a7), as well as Nz-T4 transformants carrying ECP11-1 or AvrLm3 were inoculated onto cotyledons of three cultivars carrying Rlm3 (15.22.4.1, Grizzly and Columbus), Rlm7 (15.23.4.1) or Rlm4 (Pixel). 15.23.4.1 is a sister line of 15.22.4.1 issued from individual plants from cv. Rangi, carrying Rlm7 instead of Rlm3. Pathogenicity was measured 15 days post-inoculation. Results are expressed as a mean scoring using the IMASCORE rating comprising six infection classes (IC), where IC1 to IC3 correspond to resistance, and IC4 to IC6 to susceptibility [50]. Error bars indicate the standard deviation of technical replicates.
