# Supplementary material for: A new family of structurally conserved fungal effectors displays epistatic interactions with plant resistance proteins
Source: PLoS Pathog. 2022 Jul 6;18(7):e1010664. doi: 10.1371/journal.ppat.1010664 (PMC9292093; doi:10.1371/journal.ppat.1010664)
Supplement: S4 Table — (DOC) [file ppat.1010664.s009.doc]

**Table S4: List of PCR primers used for site directed mutagenesis**

|  | **Primers  (5' – 3')** | |
| --- | --- | --- |
| *AVR47-JN3-Lo* |  | TTAAGTGTTGAGTTGCCTAAC |
| *AVR47-JN3-Up* |  | CACTAACCCTAACCTAACCTAT |
| *MD1-Up* |  | cggcgcatagatatccagaattcgttccc |
| *MD2-Up* |  | cgcatagatatcgagaatCcgttcccaaaattc |
| *MD3-Up* |  | ccctatagcagctttagAcagcacctggag |
