# Supplementary material for: A new family of structurally conserved fungal effectors displays epistatic interactions with plant resistance proteins
Source: PLoS Pathog. 2022 Jul 6;18(7):e1010664. doi: 10.1371/journal.ppat.1010664 (PMC9292093; doi:10.1371/journal.ppat.1010664)
Supplement: S1 Materials and Methods — (DOCX) [file ppat.1010664.s010.docx]

**S1 Materials and Methods: Selection of efficient protein producer clones of AvrLm5-9 and Ecp11-1**

The vector pPICZα (Invitrogen) was used to express AvrLm5-9, AvrLm3 or Ecp11-1 in *P. pastoris* without their signal peptide. The constructions contained a histidine tag (6His), Thioredoxin (Trx) and a tobacco etch virus TEV cleavage site at the N terminal part. Preparation of yeast electrocompetent cells and transformation of *P. pastoris* X33 were performed as described by the supplier (Invitrogen, K1710-01). pPICZαA-6His-Trx-Tev-AvrLm5-9, pPICZαA-6His-Trx-Tev-AvrLm3 and pPICZαA-6His-Trx-Tev-Ecp11-1 plasmids were extracted from *E. coli* TOP10F’ and digested with *Sac*I. The linear plasmids DNA (5 μg) were transformed into 100 μl of competent X33 cells by pulsed electroporation using a BTX electro cell manipulator (1,500 V, 25 µF, 200 ohms). Transformed cells were incubated on selective YEPD (10 g yeast extract, 20 g peptone, 20 g dextrose and 20 g bacto-agar in 1 liter) agar plates (zeocin™ 100 mg/L) at 30°C for 3-4 days until colonies appeared. Putative multi-copy recombinants were screened by inoculation of 3 µL of each zeocin resistant clones on increasing concentrations of Zeocin™ (500, 1000, and 2000 μg/ml Zeocin™) in the YEPD agar plates. Integration of the gene of interest into the yeast genome was confirmed by direct PCR screening of selected *Pichia* clones in using the 5 ́AOX1 and 3 ́ AOX1 primers. Protein expression levels of the selected transformants were explored by cultivation in multiplates-24 wells with 2 mL of buffered methanol complex BMMY medium (10 g yeast extract, 20 g peptone, 100 mM potassium phosphate pH 6, 400 µg biotin, 13.4 g yeast nitrogen base without amino acids and addition of 1%(v/v) methanol 1% twice a day) for 96 hours at 22°C in thermomixed comfort (500 rpm). Protein productions were estimated by SDS PAGE analysis of the filtrated and concentrated supernatants (cut off 10 kDa).
