# Supplementary material for: A new family of structurally conserved fungal effectors displays epistatic interactions with plant resistance proteins
Source: PLoS Pathog. 2022 Jul 6;18(7):e1010664. doi: 10.1371/journal.ppat.1010664 (PMC9292093; doi:10.1371/journal.ppat.1010664)
Supplement: S2 Materials and Methods — (DOCX) [file ppat.1010664.s011.docx]

**S2 Materials and Methods: Conditions for native protein production and extraction using *P. pastoris* in fed-batch cultivation**

The best secreting clones were cultivated in high cell density using a fed-batch mode of cultivation. Large-scale cultures were done in a bioreactor (DASGIP) in presence of 1 Liter of the modified buffered glycerol complex BMGY medium, containing all the components as BMGY medium except methanol is replace by 4 % (v/v) of glycerol. Ammonia 15 % (v/v) was used both to maintain the pH at 6 and as source of nitrogen. The temperature was set at 28°C, oxygenation was 1 volume of sterile air per volume of medium per minute and oxygen saturation was maintained over 10% by vigorous stirring (1200 rpm). Upon depletion of glycerol, a pulse with an identical concentrated medium was given. When the entire carbon source was consumed, induction by methanol was initiated with a gradual supply of the feed-medium. The synthetic feed-medium used for fed-batch culture contained 780 g methanol, trace elements 5X PTM1 [H_3_BO_3_ 0.1 mg, CuSO_4_.5H_2_O, 30 mg; KI 0.5 mg, MnSO_4_.H_2_O, 15 mg, Na_2_MoO_4_, 1 mg, ZnCl_2_, 100 mg, FeSO_4_.7H_2_O 325 mg, H_2_SO_4_ (96%) 0.1 mL and biotin 1 mg per liter]. The feeding profile was monitored using a computer-controlled pump to maintain the biomass yield Y_X/S_ of 0.3 g biomass per gram of methanol and a theoretical specific growth rate of about 0.015 per hour. Induction went on for three days (1.5 generation cells) after the first addition of methanol. Biomass was determined by measurement of optical density at 600 nm and dry cell weight of yeast suspensions (according to a correspondence of 0.48 g/L dry cell weight per DO_600nm_ unit).

Supernatants of the cultures were collected by centrifugation at 5000 rpm for 20 min and then filtrated (0.5 µM) and diafiltrated (10 kDa) against appropriated buffer (20 mM Tris pH 8, 300 mM NaCl, 5% v/v glycerol) by tangential flow ultrafiltration on hollow fiber cartridges (GE Quixstand system). The supernatant was used to purify the recombinant proteins.
